# Candidate Signature miRNAs from Secreted miRNAome of Human Lung Microvascular Endothelial Cells in Response to Different Oxygen Conditions: A Pilot Study

**DOI:** 10.3390/ijms25168798

**Published:** 2024-08-13

**Authors:** Wolfgang Schaubmayr, Matthias Hackl, Marianne Pultar, Bahil D. Ghanim, Klaus U. Klein, Johannes A. Schmid, Thomas Mohr, Verena Tretter

**Affiliations:** 1 Department of Anesthesia, General Intensive Care and Pain Management, Medical University of Vienna, 1090 Vienna, Austria; wolfgang.schaubmayr@meduniwien.ac.at (W.S.);; 2 TAmiRNA GmbH, 1110 Vienna, Austria; 3 Department of Thoracic Surgery, Medical University of Vienna, 1090 Vienna, Austria; 4 Department of Vascular Biology and Thrombosis Research, Center for Physiology and Pharmacology, Medical University Vienna, Schwarzspanierstraße 17, 1090 Vienna, Austria; johannes.schmid@meduniwien.ac.at; 5 Institute of Cancer Research, Department of Medicine I, Comprehensive Cancer Center, Medical University of Vienna, 1090 Vienna, Austria; thomas.mohr@meduniwien.ac.at

**Keywords:** lung endothelium, extracellular miRNA, miRNA biomarker, lung oxygen, lung biomarker

## Abstract

Oxygen conditions in the lung determine downstream organ functionality by setting the partial pressure of oxygen, regulating the redox homeostasis and by activating mediators in the lung that can be propagated in the blood stream. Examples for such mediators are secreted soluble or vesicle-bound molecules (proteins and nucleic acids) that can be taken up by remote target cells impacting their metabolism and signaling pathways. MicroRNAs (miRNAs) have gained significant interest as intercellular communicators, biomarkers and therapeutic targets in this context. Due to their high stability in the blood stream, they have also been attributed a role as “memory molecules” that are able to modulate gene expression upon repeated (stress) exposures. In this study, we aimed to identify and quantify released miRNAs from lung microvascular endothelial cells in response to different oxygen conditions. We combined next-generation sequencing (NGS) of secreted miRNAs and cellular mRNA sequencing with bioinformatic analyses in order to delineate molecular events on the cellular and extracellular level and their putative interdependence. We show that the identified miRNA networks have the potential to co-mediate some of the molecular events, that have been observed in the context of hypoxia, hyperoxia, intermittent hypoxia and intermittent hypoxia/hyperoxia.

## 1. Introduction

Since their discovery in 1993 in the worm Caenorhabditis elegans, the research field of miRNAs has hugely expanded. These small RNAs have been shown to regulate the majority of cellular gene expression in a nucleotide sequence-specific way [1]. Due to their short length (mostly between 19 and 22 nucleotides), and the even shorter seed region, a single miRNA is capable of regulating the expression of many genes thereby orchestrating complex gene expression patterns. They are often secreted from cells and can travel in circulation over longer distances, well protected either in vesicles or bound to lipoproteins, before they can be taken up again by distal tissues to also regulate gene expression in remote organs [2,3]. Transcription of miRNAs is somewhat cell-type and tissue-specific and circulating levels have been shown to have predictive potential for organ (dys)function. Samples can be obtained from a non-invasive blood collection, and miRNAs can be specifically quantified using standard analytical procedures (qPCR). Therefore, miRNAs have attracted significant interest as biomarker candidates in the context of several pathophysiological conditions, such as cancer, neurodegeneration and diseases of the heart, kidney, liver and lung [3,4]. In addition, they are regarded as promising therapeutical tools in precision medicine [5].

Acute respiratory distress syndrome (ARDS) can be elicited by different triggers, such as infection, aspiration, etc., and frequently has a fatal outcome due to its heterogenous clinical manifestation and the lack of effective therapeutic interventions. Mechanical ventilation using supplemental oxygen is necessary in many cases in order to ensure proper tissue oxygenation but can, *per se*, have detrimental impact on the lungs, including volu-, baro- and atelectotrauma. The worst danger, however, comes from biotrauma, which is still not understood in every detail. Hyperoxia induces oxidative stress and the formation of oxygen-derived radicals, as well as impacts on normal signaling pathways, that can be regulated dependent on the partial pressure of oxygen. Impaired and mechanically ventilated lungs are further prone to forming atelectasis resulting in oxygen patterns that oscillate during the breathing cycle between hypoxic and hyperoxic levels. In animal models, it has been shown that these oxygen oscillations are transmitted via the circulation to remote organs and might have pathophysiological consequences [6]. This condition of intermittent hypoxia/hyperoxia has been shown to result in the activation of biological pathways different from constant hypoxia or hyperoxia [7]. Oscillations in the hypoxic range are observed in obstructive sleep apnea syndrome (OSAS), commonly designated as “intermittent hypoxia”, which underlies part of the pathophysiology of the condition. Apart from reactive oxygen species (ROS) miRNAs might also be involved in regulating these effects in an auto-, para- or endocrine way. Therefore, in this study, we primarily aimed to analyze miRNAs secreted from human lung microvascular endothelial cells in a well-defined culture system in response to different oxygen conditions. We aimed to predict potential effects on nearby or remote cells by using their known target genes in enrichment analyses and tested possible autocrine effects in their mother cells. The identification of miRNAs in the lung endothelial secretome as a signature for diverse oxygen conditions could help to delineate molecular effects on target mRNAs, elucidate potential impact of oxygen conditions on lung injury in the context of pathological states such as ARDS or OSAS and could enable us to propose candidate miRNAs that could serve as biomarker (panels) originating from the lung endothelium.

## 2. Results

### 2.1. Workflow

**Figure 1:**
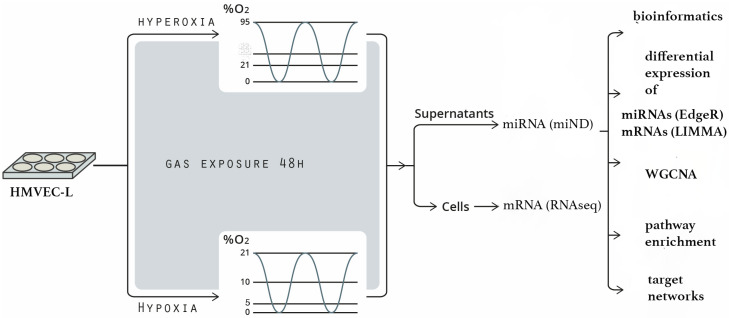
Workflow showing HMVEC-L exposure to different O_2_ conditions, biochemical processing, RNA sequencing and bioinformatic processing of data.

Human lung microvascular endothelial cells (HMVEC-Ls) from six supposedly healthy Donors (but unknown anamnesis) were exposed to constant and oscillating oxygen (O_2_) conditions. Oxygen conditions were chosen to represent moderate and mild hypoxia (5% and 10% O_2_), strong hyperoxia (95% O_2_) and intermittent hypoxia (0–21% O_2_) or intermittent hypoxia/hyperoxia (0–95% O_2_) relative to normoxic culture conditions (21% O_2_ = reference condition in all calculations). Supernatants were concentrated via ultrafiltration in order to enrich them for vesicle- or lipid-bound miRNAs. miRNAs in concentrates and mRNAs in lysates of mother cells were analyzed via sequencing followed by bioinformatic data analyses (Figure 1). The aim of this study was to possibly identify O_2_ condition-specific changes in secreted miRNAs and their putative autocrine or para- and endocrine effects.

### 2.2. Characterization of Secreted miRNA during Different Oxygen Conditions

Analysis of secreted miRNAs (primarily from extracellular vesicles, but also lipid-bound) was performed using clarified and concentrated conditioned medium from HMVEC-Ls.

Prior to next-generation sequencing of miRNAs, 42 miRNAs were compared via qPCR to identify a potential impact of concentration via ultrafiltration on miRNA levels. The data showed that most miRNAs were increased in concentration by a mean factor of about 12.5, indicating uniform recovery of miRNAs after ultrafiltration. Spike-in controls also indicated an absence of PCR inhibition after ultrafiltration, which was relevant for additional miRNA quantifications via qPCR (Supplemental Data S1A).

**Figure 2:**
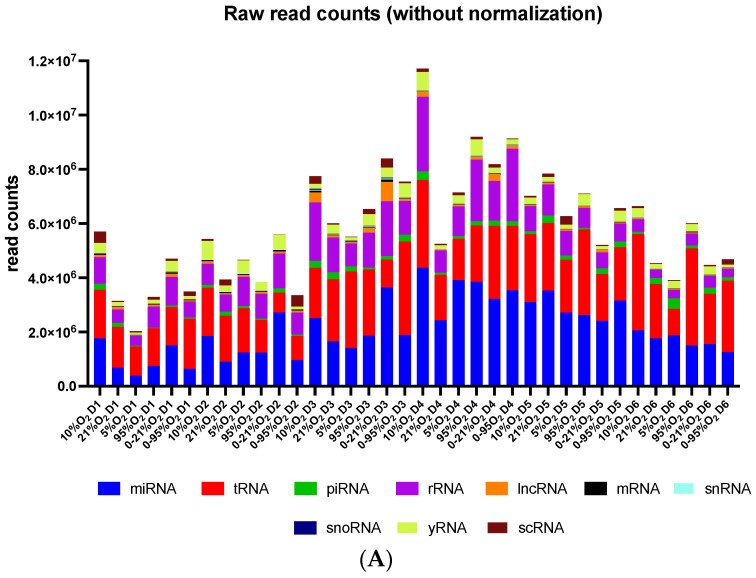

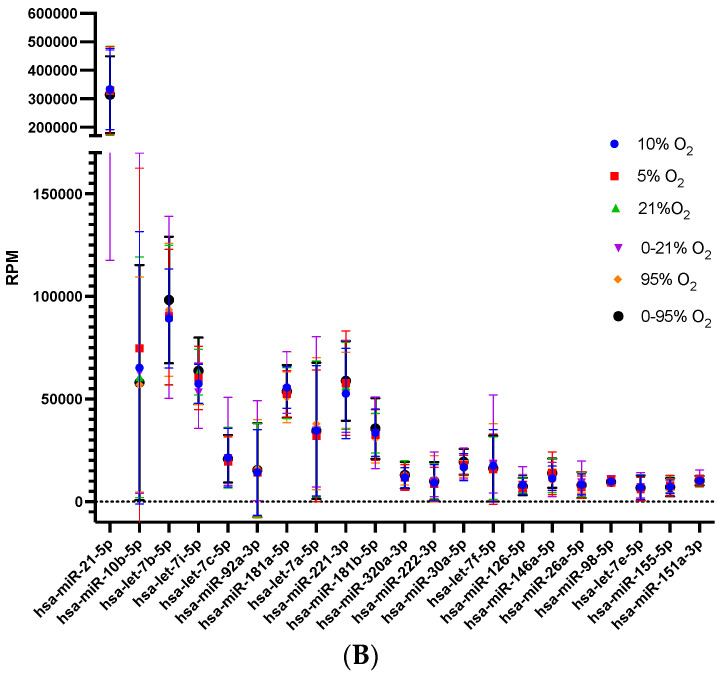
Visualization of data from next-generation sequencing of secreted extracellular (mi)RNAs. (**A**) Quantitative distribution of different types of RNAs detected in individual samples as obtained from raw reads without any further normalization. (**B**) Top 21 miRNAs with highest abundance (shown as mean and range from 6 Donors in “Reads per Million”-RPM).

The hierarchical mapping approach of quality- and size-filtered sequencing of raw data against various transcriptome databases revealed comparable RNA composition across all samples despite differences in absolute read counts (Figure 2A). Figure 2B shows the 21 top abundant miRNAs in supernatants, and the whiskers indicate variability between the six Donors from this study. A heatmap created from data of all detected miRNAs and principal component analysis reveal strong Donor-specific characteristics (Supplemental Data S1B).

### 2.3. Identification of Secreted miRNA Subsets in Response to Different Oxygen Conditions

**Figure 3:**
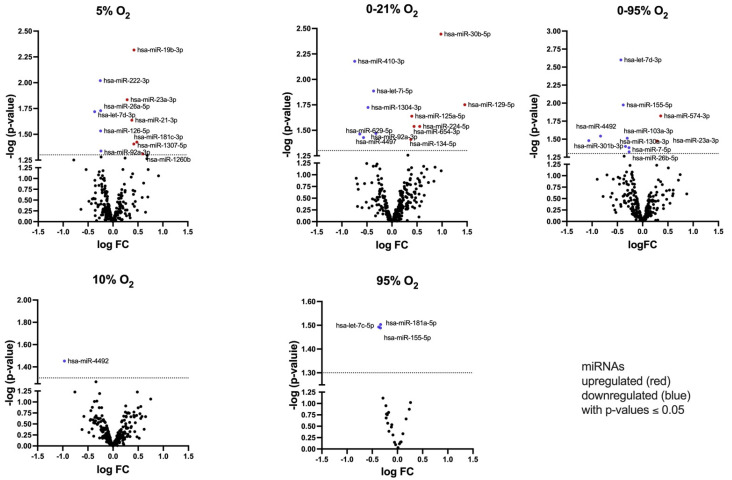
Volcano plot showing log (fold change) of miRNA abundance relative to uncorrected *p*-value. Shown are all miRNAs with 0.05 < *p*-value < 1.0 as black dots. The dotted line represents a threshold of *p* = 0.05. miRNAs with *p* < 0.05 are labeled and assigned blue (downregulated) or red (upregulated) symbols.

Differential expression of miRNAs in different groups (with 21% O_2_ as reference treatment) was investigated using edgeR. We obtained a list of miRNAs, that were changed with *p*-values < 0.05 relative to normoxic incubation; however, after corrections for multiple testing according to the Benjamini–Hochberg procedure, only hsa-miR-181b-5p achieved an FDR <0.1 in the 95% O_2_ treatment group (Table 1). In addition to a Donor effect, which was identified using unsupervised analysis (Supplemental Data S1B—Heatmap, PCA and t-SNE [8]), treatment-induced changes in miRNA abundance usually are subtle, and a biological impact is mainly achieved through cooperative networks of several miRNAs. We therefore selected miRNAs with *p* < 0.05 (graphically depicted in Volcano plots, Figure 3) and extended the number of Donors for verification of some miRNAs (top 3–5 miRNAs with smallest *p*-value from NGS) via qRT-PCR (Table 1).

Table 2 lists the role of individual identified miRNAs in signaling pathways as deduced from the web-platform miRNet (www.mirnet.ca; accession date 15 March 2024) for enrichment analysis of target genes.

In order to identify potentially more robust miRNA hits, we used the R-package LIMMA as an additional data analysis approach. Originally developed for the detection of differentially expressed genes in microarrays, LIMMA has developed as a widely used standard in the analysis of transcriptomics and proteomics data in experiments with complex experimental designs. Briefly, LIMMA fits a regression-like linear model to the data and experiment design, thus allowing the estimation of the logFC, the *p*-value and the adjusted *p*-value. LIMMA analysis was followed by weighted gene coexpression network analysis (WGCNA) according to Langfelder and Horvath [9]. Briefly, in WGCNA, coexpression networks are calculated using Pearson’s correlation coefficient. These coexpression networks are segmented into groups of genes (modules) via hierarchical clustering of the coexpression matrix and cutting the resulting dendrogram using dynamic tree cutting. Thus, groups of genes with similar expression patterns are obtained, which can be put into biologic context using term enrichment analysis. WGCNA reduces the problem of post hoc correcting of data for multiple testing. Highly interconnected genes are clustered in “modules” to summarize genes with high absolute correlations. Characteristics of such networks are the following: (1) the “connectivity” (or “degree”), which is a measure for the connection strength with other network genes; (2) the “intramodular connectivity k_within_”, which is a measure for connectivity of a gene to the genes of a particular module; (3) the module “eigengene”, which is the first principal component of a module and therefore the main representative of the gene expression profile in this module; (4) the “eigengene significance”, which is the correlation coefficient of the module eigengene with the outcome (sample trait); (5) the “module membership” (correlation of the expression profile of a gene with the module eigengene of a given module); (6) “hub genes” representing genes inside modules with high connectivity; (7) “gene significance”, which provides a measure of how significant a certain gene is for the biological question and (8) “module significance”, which is the average gene significance of all genes in a given module.

In summary, the workflow comprises the construction of a gene coexpression network via correlation analysis, the identification of modules through hierarchical clustering, putting modules in relation to biological information, reduction of biological data to eigengenes and the identification of key drivers in interesting modules (intramodular connectivity) in order to identify biomarker candidates.

**Figure 4:**
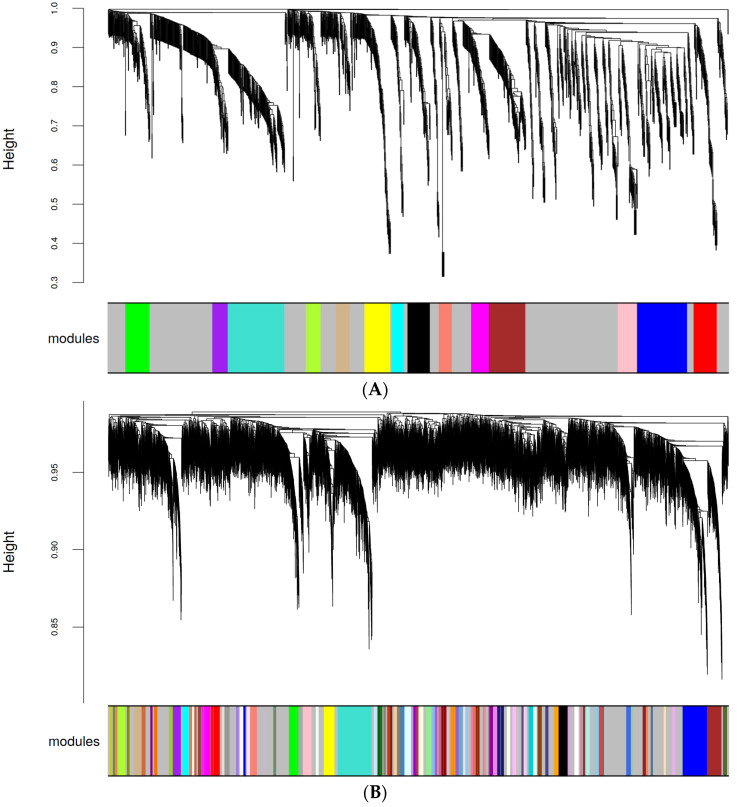
(**A**) WGCNA of miRNA identified and quantified via NGS in the HMVEC-L secretome. Clustering tree (dendrogram) generated through hierarchical clustering of adjacency-based dissimilarity detected modules with similar coexpression. The dendrogram and module distribution shows that the data could be cleanly divided into groups of genes (modules) indicating a well-defined structure of the coexpression network. Modules are color-coded. The gray module combines all remaining miRNAs with no coexpression behavior. (**B**) WGCNA of mRNA quantified via RNAseq in the secreting HMVEC-L (mother) cells. Clustering tree (dendrogram) generated by hierarchical clustering of adjacency-based dissimilarity detected modules with similar coexpression. Modules are color-coded. The gray module combines all remaining mRNAs with no coexpression behavior.

The WGCNA of our data from secreted miRNAs in this study resulted in 15 modules (M0-M14) with *p*-values not below 0.05 (Table 3; Figure 4A), which certainly limits any valid interpretation. Underlying causes are the small sample size (due to limited availability of different Donor cells with appropriate growth characteristics) and the rather small treatment effect on levels of individual secreted miRNAs. In order to deduce a possible trend that can be a starting point for further studies, we make an interpretation attempt: modules exhibiting correlations with the lowest *p*-value were M8 (k = 0.393; *p* = 0.149 for 5% O_2_; k = −0.293; *p* = 0.295 for 0–21% O_2_ oscillations), M13 (k = 0.358; *p* = 0.193 for 0–95% O_2_ oscillations), M14 (k= 0.324; *p* = 0.243 for 95% O_2_), M0 (k = −0.271; *p* = 0.334 for 0–21% O_2_) and M2 (k = −0.251; *p* = 0.372 for 0–21% O_2_). Interestingly, we found the miRNAs from the alternative arm of the NGS-identified precursor miRNAs let-7i-3p, miR-1304-5p, miR-125a-3p, miR-224-3p, miR-629-3p, miR-134-3p and miR-129-1-3p in modules M0 and M2 for 0–21% O_2_. Correlation coefficients and *p*-values of all modules are shown in Table 3.

In order to predict putative biological processes mediated via miRNA-targeted genes, we performed enrichment analysis of miRNA targets from individual modules. Detailed results from the enrichment analysis are listed in Supplemental Data S2. Items with low *p*-values represent possible candidates as a basis for further mechanistic studies.

Table 4 shows connectivity values of miRNAs central in the respective modules, indicating especially important miRNAs for the processes represented by the module.

### 2.4. RNA Sequencing from the Mother Cells of the Secretome

RNA sequencing was primarily performed for the secreted miRNAs, but also for mRNAs of the mother cells (HMVEC-Ls) from three Donors under the same O_2_ exposure condition. This was realized in order to get an estimate of gene expression regulation on the level of the mother cells. Cellular miRNA levels will be different from secreted miRNAs, but secreted miRNAs can also influence their mother cells (=autocrine effect). The autocrine effect of secreted miRNAs, however, is difficult to dissect from intrinsic miRNA- and other regulative mechanisms of mRNA expression and stability, but we attempted to deduce possible parallel tendencies.

According to data analysis of secreted miRNAs as described above, LIMMA–WGCNA was applied to the RNAseq data from cell lysates resulting in 95 modules with similar coexpression (Figure 4B). Correlations of all modules are shown in Table 5.

The modules of cellular mRNAs exhibiting statistically significant positive or negative correlations with O_2_ conditions are highlighted in Table 5.

Detailed data from the enrichment analysis of all modules are extensive and are therefore shown in Supplemental Data S3. Modules with strongly significant correlations (*p* < 0.0001) and their key enriched biological processes (GOBP terms) are summarized in Table 6.

### 2.5. Comparison of Cellular Signaling in Response to O_2_ Oscillations with Different Amplitudes

It is especially interesting to use these data to compare molecular mechanisms between oxygen oscillations with the same frequency but different amplitudes (0–21% O_2_ and 0–95% O_2_). Outcome parameters from previous studies from our research group have indicated that molecular signaling pathways most likely are different in several aspects [7,10]. The structures of modules with significant *p*-values (as indicated by correlation coefficients) are overlapping in some aspects but also reversed in other details (see Table 5). Generally, stronger correlations with higher statistical significance are observed in the samples exposed to 0–21% O_2_ compared to 0–95% O_2_ indicating a stronger expression of trait. Modules with significant positive correlation in both oscillation treatments (M47, M77 and M80) are associated with SMAD and BMP signaling, negative regulation of TGFβR pathway, cytoprotection by HMOX1, fucosylation, HIF1 targets, organization of cell–cell junction, positive regulation of exocytosis and excretion (including extracellular vesicles), glycolysis and gluconeogenesis, as well as glucose metabolism. Modules with significant negative correlation in both oscillation treatments (M14, M32, M42 and M66) are associated with GPI anchor biosynthesis, ceramide pathway, regulation of cell–cell adhesion, PTEN pathway, metabolism of porphyrins, nucleotide excision repair, double strand break repair via NHEJ, positive regulation of receptor mediated endocytosis, programmed cell death, protein targeting to peroxisomes. Positive correlation in 0–21% O_2_ treatment and negative correlation in 0–95% O_2_ treatment (M8, M54, M84 and M91) are associated with regulation of ROS biosynthesis, cytochrome complex assembly, degradation of extracellular matrix, TNFR2 non-canonical NF-κB pathway, mitochondrial respiratory chain complex assembly, cytokine production, calcium signaling using intracellular calcium, cell–cell communication, regulation of the nitric oxide metabolic process, positive regulation of stress fiber assembly, reactive nitrogen species (RNS) metabolic process, interferon-gamma production, IL-1, IL-6, IL-12 and TNFα (generally cytokine) production, positive regulation of TLR and pattern-recognition receptor signaling pathway and collagen degradation. Negative correlation in 0–21% O_2_ treatment and positive correlation in 0–95% O_2_ treatment (M7, M22, M23, M56 and M72) are associated with morphogenesis (tube formation), cell division and mitotic cell cycle, positive regulation of TORC1 and TOR signaling, positive regulation of response to DNA damage stimulus, negative regulation of extrinsic apoptotic signaling pathways, ribosome and translation initiation, response to starvation, the PDGF pathway, telomere maintenance via telomere lengthening, cellular senescence, lipid modification, fatty acid metabolism and protein localization to the ER.

### 2.6. Putative Autocrine Effects of Secreted miRNAs on Cellular mRNA Targets

The in vitro system of this study uses only one cell type (HMVEC-L); therefore, target cell effects of secreted miRNAs can only be directly referred to the mother cells (=autocrine effect). In order to relate changes in miRNA levels in the secretome to alternated mRNA levels of putative miRNA targets in the cells, we applied iTALK, an open source R package that can be used to characterize and illustrate intercellular communication: https://github.com/Coolgenome/iTALK (Accessed on 7 March 2024) [11,12]. The software was designed to visualize ligand–receptor-mediated intercellular cross-talk but can also be adapted to miRNA–mRNA interactions from sequencing data.

**Figure 5:**
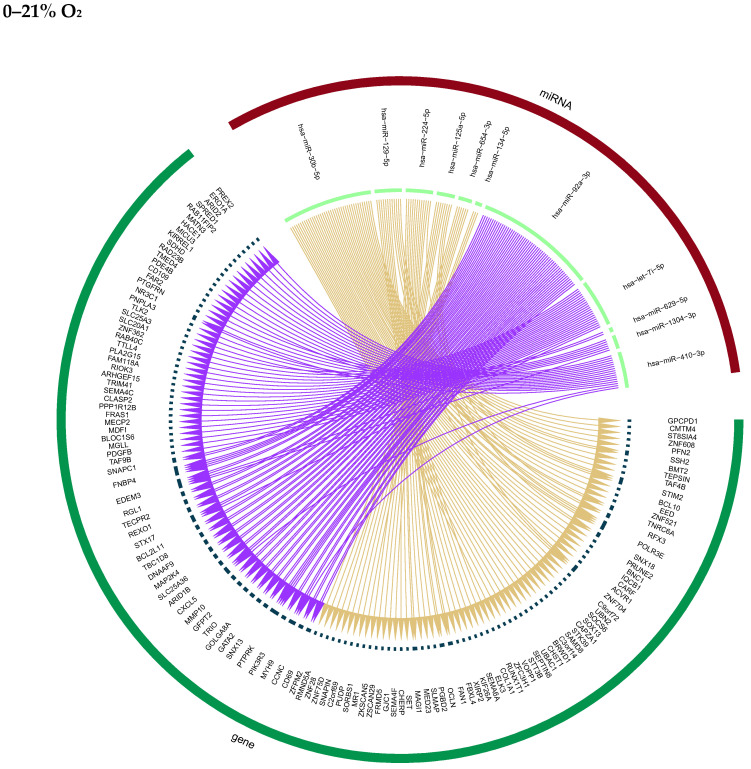

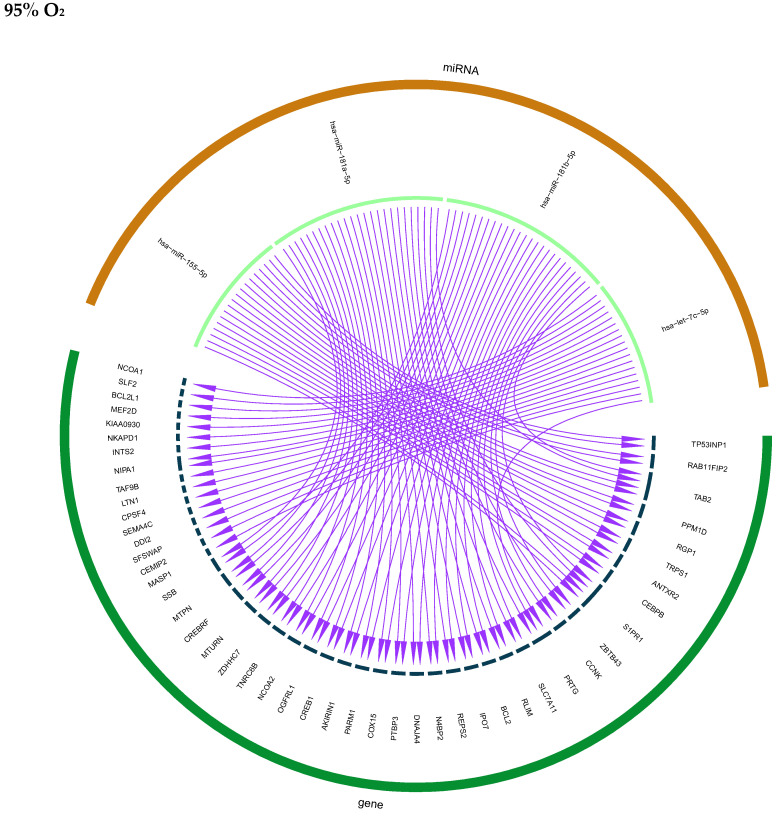
Visualization of iTALK outputs as Circos plots. miRNAs with altered abundance in the secretome after 48 h treatment with 0–21% O_2_ and 95% O_2_ are plotted and put into relation with putative target genes from cellular RNAseq data with purple arrows indicating downregulated miRNAs and upregulated target genes and beige arrows linking upregulated miRNAs and the respective downregulated mRNA targets.

Output data were filtered and ranked according to q-value and interaction pairs showing an inverse expression trend between miRNA and putative target mRNA were visualized in Circos plots. Clear trends were only observed with treatment groups of 0–21% O_2_ and 95% O_2_ (Figure 5). Identified target genes were analyzed with the Reactome database (www.reactome.org, Accessed on 7 March 2024) in order to deduce potential autocrine effects (Table 7).

### 2.7. Putative Para- and Endocrine Effects

**Figure 6:**
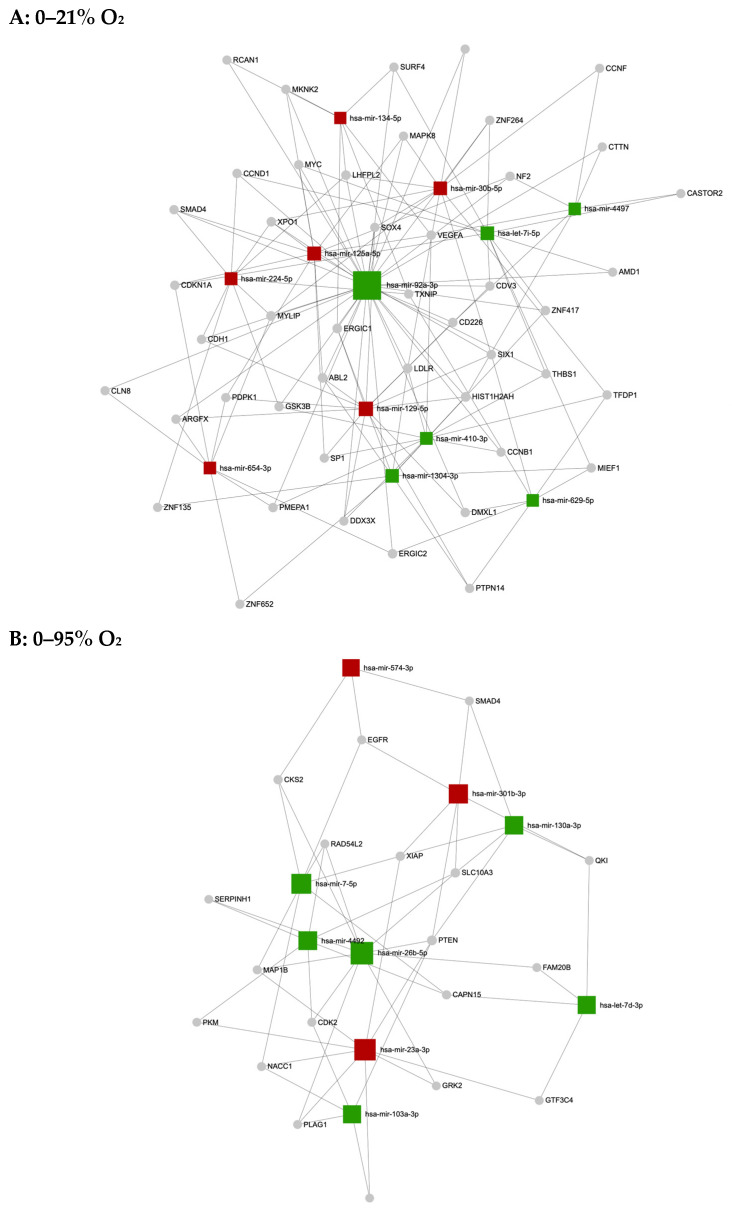
Secreted miRNA–target gene interaction networks under (**A**) 0–21% O_2_ and (**B**) 0–95% O_2_ oscillations generated with software miRNet 2.0 (www.mirnet.ca). Upregulated miRNAs are highlighted in red; downregulated miRNAs are highlighted in green.

In order to visualize differences of secreted miRNA/target mRNA interactions under O_2_ oscillations with different amplitudes, we constructed secreted miRNA–putative target mRNA networks using www.mirnet.ca software miRNet 2.0 for 0–21% O_2_ and 0–95% O_2_ oscillations (Figure 6).

Enrichment analysis of target genes in such networks for constant and intermittent O_2_ conditions was performed in order to predict possible biological effects that might be induced in other target cells via cooperative action of their associated miRNAs (Table 8).

## 3. Discussion

The original purpose of our study was to aim for the identification of marker miRNAs that are secreted from the human pulmonary microvasculature in response to different oxygen conditions or patterns and play a role in the clinical setting, such as different hypoxic conditions as in tumors or due to O_2_ undersupply, hyperoxia elicited by supplemental O_2_ therapy, intermittent hypoxia as in OSAS or also in tumors and intermittent hypoxia/hyperoxia due to atelectasis in ventilated patients with higher FiO_2_. Influencing factors in this context are highly complex: Donor characteristics such as age, gender, ethnicity and confounding factors (co-morbidities, smoker and environmental factors), cell type, duration of gas exposure and timepoint of sampling and oxygen concentration, just to name a few. The task becomes even more difficult if we want to conclude downstream effects of such specific signature miRNAs, as they will have multiple potential target mRNAs engaging in a plethora of sometimes even contrasting signaling pathways. miRNAs secreted into the circulation may be taken up by cells of nearby or remote tissues, and it is still unclear if these uptake mechanisms are highly specific or partly promiscuous. Bioinformatic analysis methods help to sort extensive data, portray an overall picture of the setting and can contribute to narrowing the hypothesis.

In our pilot study, we tried to simplify the system as much as possible. We chose as a model system pure preparations of supposedly healthy human pulmonary microvascular endothelial cells in culture from Donors of different gender and ethnicity, in order to identify treatment-specific effects independent from the individual. These Donor cells were exposed in parallel to the different O_2_ conditions for 48 h. Exposure duration was chosen to achieve the best trade-off between the amount of secreted miRNAs (protein-bound or in extracellular vesicles-EVs) and stability of EVs.

Pure preparations of HMVEC-Ls from healthy human lungs of sufficient cell counts to conduct these experiments represent very precious material and availability is very limited. It is generally difficult to obtain effects with statistical significance after post hoc corrections (Benjamini–Hochberg) when testing for hundreds of individual items (=miRNAs in NGS), especially because several miRNAs usually show small to moderate changes upon treatment but act in cooperation to alter downstream mRNA expression resulting in biological effects.

As part of a pilot study, our results were extracted from the NGS analysis of six Donors and a fraction of miRNA alterations could be verified in a further six Donors via qRT-PCR. Data analysis and prediction of potential functional implications were performed with the help of bioinformatic methods. Most identified extracellular miRNAs have been annotated to occur in extracellular vesicles and some miRNAs have been already described in the literature in the context of endothelial function (for example: miR-92a, miR-126, miR-98, and miR-181). Many miRNAs have been mainly investigated in the context of cancers; therefore, the role of these miRNAs in non-cancerous cells needs to be interpreted with caution.

The exact experimental condition is crucial for the interpretation of the outcome. The literature contains a large number of different studies that have investigated, for instance, hypoxia-regulated miRNAs (HRM). Their significance, however, is strictly dependent on the cell type, the actual O_2_ concentration and the duration of exposure. Central regulators of the hypoxic cell response are a family of hypoxia-inducible transcription factors (HIFs). Part of HIF-dependent signaling is regulated by various miRNAs, such as HIF biogenesis, activation, degradation and isotype-switch and translation of HIF-dependent proteins [13]. Moreover, 5% O_2_ is relatively moderate but not severe hypoxia and might be above the threshold of induction for hypoxia-induced transcription factors of the HIF family. HIF1A is even a target gene of the upregulated miR-19b, as well as the DNA-methyltransferase DNMT3A, which regulates DNA methylation under hypoxia. miR-19b together with miR-23a has been identified as a hub miRNA linking hypoxia and DNA methylation in thrombotic conditions [14]. We identified miR-19b-3p and miR-23a-3p as miRNAs with high intramodular connectivity (k_within_) in M1 in WGCNA, which also reveals their function as hub miRNAs in this module. miR-19b also targets PTEN, a negative regulator of the Akt-pathway and thereby enhances proliferation and inhibits apoptosis, which is a common observation under mild hypoxia. Similarly, miR-21 (in M3) targets PTEN and impacts DNA methylation [15]. Downregulation of the tumor suppressor miR-26a and let-7 family member let-7d-3p will further support cell proliferation [16]. miR-181c has been associated with COPD [17]. miR-1307 is an oncogenic miRNA associated with proliferation and metastasis and has been shown to play an important role in cellular infection by SARS CoV-2 virus and disease progression [18]. miR-92a belongs to the miR-17-92 cluster and is used as exosomal biomarker in several cancers. Levels of miR-92a in exosomes can indicate the level of activation of the endothelium and endothelial dysfunction. In cardiovascular medicine it is used as a marker for the distinction of acute myocardial infarction and stable coronary heart disease [19]. Pathway analysis of these extracellular miRNAs revealed their active function in a typical and previously described hypoxic response. Enriched pathways of miRNAs secreted under 5% O_2_ (= moderate hypoxia) included the following: TGFß and VEGF signaling as a typical hypoxic response [20], ATF4 induces VEGF-A and accumulates in ER under hypoxia [21], increased cell proliferation supported by decreased cellular senescence and DNA damage/telomer stress, a tendency to pro-thrombotic events via fibrin clot formation [22], decreased collagen degradation in the ECM [23], smooth muscle contraction (possibly as part of the pulmonary hypoxic vasoconstriction), hypoxic p53 signaling [24], calcium-activated K+ channels [25], glucose transporter translocation [26] and regulation of lipid metabolism [27]. Other miRNAs that are frequently named in the context of hypoxic regulation, such as miR-210, miR20a/b, miR-199a, miR-200b/c, miR-424 and miR-429, did not stand out in this context, either because they strongly varied between Donors (i.e., hsa-miR-424 and hsa-miR-199a) or reads were extremely low or zero in the secretome (i.e., hsa-miR-20a/b, hsa-miR-210, hsa-miR-200b/c and hsa-miR-429).

Furthermore, 10% O_2_ is an even milder hypoxic condition and revealed only a downward trend of miR-4492, possibly reducing cellular senescence.

Additionally, 95% O_2_ is a very strong hyperoxic condition that induces cell stress, DNA damage by ROS, inflammation via NF-KB activation and senescence. ROS mainly occurs in the mitochondria and through NADPH oxidase (NOX). The miRNA-181a and 181b couple target several proteins involved in mitochondria biogenesis, turnover and clearance as well as detoxification of ROS. Downregulation of miR-181a and 181b has proven to ameliorate disease conditions associated with mitochondrial dysfunction, such as retinal diseases, where oxidative stress is an issue [28]. miR-155 has a critical role in oncogenesis, inflammation and immunity [29].

The 0–21% O_2_ condition, or intermittent hypoxia, is a model for oxygen conditions occurring in tumors or in obstructive sleep apnea (OSAS) and has been shown in the literature to activate very specific signaling pathways. miR-30b-5p has been shown to play an important role in the suppression of lysosmal biogenesis and autophagy [30]. It is a tumor suppressor in the context of lung cancer and enhances apoptosis [31]. Experimentally validated targets are CCNE1, SOCS1, SMAD1, CAT and BCL6 indicating a role in the regulation of cytokine signaling, apoptosis and oxidative stress. Validated targets of miR-129-5p are CAMTA1 (calmodulin-binding transcription factor), indicating a role of calcium under this condition, SOX4 (regulation of Wnt pathway), GALNT1 (modifying glycan structures), EIF2C3 and HMGB1, which has been shown to protect against ischemia/reperfusion injury [32]. Apart from the enriched pathways that have miR-125a-5p in common with other miRNAs, an interesting finding comes from the literature. Hirota et al. showed that miR-125a-5p and its family member miR-125b-5p target ATRAP (AT1R-associated protein), thereby activating angiotensin II-AT1R signaling and facilitating hypertension, which is also a phenotype in OSAS [33]. miR-654-3p was found to reduce inflammation by targeting ADAM10 and RAB22A (both promoters of atherosclerosis) [34] and TNFRSF9 (TNF receptor family member 9) [35]. Activated NF-KB can upregulate miR-224-5p [36], which was shown to be a circulating indicator of patients’ responsiveness to glucocorticoid therapy as it downregulates glucocorticoid receptor degradation by inhibiting GSK-3ß [37]. miR-134-5p has been shown to play a role in the regulation of vascular smooth muscle cell phenotypic switch, vascular development, platelet activation, regulation of the excretion of matrix metalloproteinases of the ADAMTS family, inflammation and calcium signaling. Overexpression seems to suppress pathological vascular remodeling [38]. Downregulated miRs under 0–21% O_2_ were miR-410-3p, let-7i-5p, miR-92a-3p, miR-4497 and miR-629-5p. Downregulation of miR-410-3p has been shown to enhance adipogenesis mediated via its target IRS-1 (insulin receptor substrate-1) [39]. Intermittent hypoxia is a condition of enhanced oxidative stress. Li et al. have shown that exosomes released from endothelial cells under oxidative stress reveal downregulated miR-92a-3p, which might stimulate angiogenesis via its target gene Tissue factor [40]. In a previous study, our group was able to show that Tissue factor expression was enhanced under intermittent hypoxia and was increasingly released within extracellular vesicles [41]. Direct targets of miR-4497 are NF-KB2 and SP1 transcription factor. Therefore, down regulated miR-4497 might enhance inflammation, apoptosis and the DNA damage response [42]. miR-629-5p has been shown to be upregulated in the plasma of patients with pulmonary arterial hypertension and also in hypoxic pulmonary artery smooth muscle cells, promoting vascular remodeling via FOXO3 and PERP [43]. Intermittent hypoxia seems to induce downregulation of miR-629-5p.

Hypoxic/hyperoxic oscillations (0–95% O_2_) have been shown in previous studies to use different signaling pathways than hypoxic oscillations or constant hypoxic or hyperoxic conditions [7,44]. We found, as exosomal miRNAs upregulated under this condition, miR-574-3p, which has been shown to be linked to HIF/VEGF signaling at least under hypoxia [45], and miR-23a-3p (upregulated as under 5% O_2_). miR-155-5p was similarly downregulated as under constant strong hyperoxia (95% O_2_). Let-7d-3p was downregulated analogous to hypoxic (5% O_2_) treatment. An interesting target of identified miR-4492 is CAPON (nitric oxide synthase 1 adapter protein), which interacts with NOS1 and plays a role in psychiatric as well as heart diseases. Downregulated miR-4492 might increase CAPON expression, which is linked to induction of cardiac disease (sudden cardiac death) [46]. CAPON also plays a role in diabetes. It has a functional role in NOS1 anchoring and activation. In a previous study from our group, we found increased levels of nitric oxide-derived peroxynitrite in pulmonary endothelial cells exposed to hypoxic/hyperoxic oscillations [7]. Whether this is due to altered NO production and/or oxidative stress has not been delineated yet. miR-301b-3p plays a role in different types of cancers. Functional enrichment of oxygen-dependent proline hydroxylation points to a role of HIF regulation, similar as with miR-130a-3p. Downregulation of miR-130a-3p seems to have a pro-inflammatory effect on endothelial cells [47]. miR-7-5p targets are experimentally well validated and include PAK1, EGFR, IRS, RAF, CKAP and others. Functional studies of altered expression have mostly been conducted in the context of tumors. miR-98-5p levels have been found to be decreased in the serum of stroke patients and mice with ischemic conditions. Yu et al. found that miR-98-5p protects against oxidative stress (possibly also in the context of I/R injury) and inhibits apoptosis [48].

Analysis of mRNA expression in secreting mother cells was limited to a still smaller sample size (three Donors) but revealed molecular characteristics that are known to be associated with the respective O_2_ condition. An attempt was made to correlate changes in mRNA levels as targets of altered secreted miRNAs (= a possible autocrine effect). In samples from 0–21% O_2_ and 95% O_2_, we could identify target mRNAs of altered miRNAs in the secretome that were inversely regulated. A true autocrine effect cannot be discerned from independent effects of cellular miRNAs regulating expression of cellular mRNAs. However, a recent study shows that endothelial cells are capable of reprograming vascular cells via their EV-cargo (miRNAs and proteins) which can have different apical and basolateral loading mechanisms [49].

Again, a major limitation of our study is the small number of different Donor cells due to a very limited availability of healthy human lung tissue. This fact restricts the statistical power of this study, and, additionally, relatively small treatment effects impede the identification of miRNAs that could serve as O_2_-condition-specific markers from the lung microvascular endothelium. However, some identified miRNAs can be regarded as candidates and should be validated in further studies in the future.

## 4. Materials and Methods

### 4.1. Cell Culture

Primary human lung microvascular endothelial cells were purchased from LONZA (HMVEC-L cat.nr. CC-2527) and were propagated in EGMTM-2MV microvascular endothelial cell growth medium-2 including growth factor supplements (cat.nr. CC-3202) (LONZA, Basel Switzerland). For details on Donor characteristics (age, gender and ethnicity) see Supplemental Data S5. For the experiments, cells were plated into gelatine-coated 6-well cell culture plates with a gas-permeable fluorocarbon membrane as growth support (“Imaging Plates”, Zellkontakt, Nörten-Hardenberg, Germany) using full growth medium. Prior to gas exposure, the medium was replaced with 2 mL/well serum-free EGMTM-2MV medium.

### 4.2. Exposure to Oxygen Conditions

Gas exposure was performed in our custom-designed exposure boxes, where culture plates are supplied with gas from gas outlets underneath the individual wells as described in [50]. We used constant and oscillating oxygen conditions as follows: 5% O_2_, 5% CO_2_, rest N_2_; 10% O_2_, 5% CO_2_, rest N_2_; 21% O_2_, 5% CO_2_, rest N_2_; 40% O_2_, 5% CO_2_, rest N_2_; 95% O_2_, 5% CO_2_; 0–21% O_2_ oscillations, 5% CO_2_, rest N_2_ and 0–95% O_2_, 5% CO_2_, rest N_2_. Oscillations were produced with an electronic switch, which alternately opens the valves of two gas bottles containing the respective amplitude gas concentrations in 6 min intervals. The electronic switch was custom designed using EL-flowmeter controllers (Bronkhorst, AK Ruurlo, The Netherlands).

### 4.3. Processing of Cell Culture Supernatants

After 48 h, gas exposure cell culture supernatants were collected and were centrifuged for 10 min at 200 g to remove cells and larger debris and for 10 min at 2000 g to remove smaller parts such as apoptotic bodies. Enrichment of extracellular vesicles was achieved via ultrafiltration using Ultra-15 filters (30 kDa, MERCK cat.nr. UFC903024, miRNet 2.0) at 3200 g. Supernatants were concentrated from 12 mL to a final volume of 500 μL. Then, 200 μL concentrate was mixed with 1 mL Qiazol lysis reagent (Qiagen, Hilden, Germany) and left at room temperature for 10 min and then frozen at −70 °C. Additionally, 300 μL were directly frozen at −70 °C in low-binding tubes without prior lysis (Eppendorf, Hamburg, Germany) until further processing.

### 4.4. RNA Isolation for Next-Generation Sequencing

Total RNA extraction was performed from 200 µL ultrafiltrate using the miRNeasy mini kit (Qiagen, Hilden, Germany). Synthetic oligonucleotides obtained from the miRCURY Spike-In kit (Qiagen, Hilden, Germany) were added to the Qiazol lysis buffer before homogenization. Glycogen (5 mg/mL) was added to the chloroform extract at a 1:100 dilution to enhance precipitation. All other steps were performed according to the recommendations of the manufacturer. Total RNA was eluted in 30 µL nuclease-free water and stored at −80 °C in low-binding tubes until further analysis.

### 4.5. Small RNA-Sequencing Analysis (Next-Generation Sequencing)

Equal volumes of total RNA were used for small RNA library preparation using the CleanTag library preparation kit (TriLink Biotechnologies, San Diego, CA, USA). Adapter-ligated libraries are amplified using barcoded Illumina reverse primers in combination with the Illumina forward primer. Pools of 16–24 smallRNA sequencing libraries were prepared at equimolar rates on the basis of DNA-1000 high-sensitivity bioanalyzer results (Agilent, Santa Clara, CA, USA). Sequencing was performed on an Illumina HiSeq 2500 System with 50 bp single-end runs. Sequencing Reads were adapter trimmed and filtered for low-quality reads (Q < 30). MicroRNA annotation was performed using the miND pipeline [51,52].

### 4.6. Quantitative Real-Time PCR

For qRT-PCR, RNA was isolated using the miRNeasy Mini kit (Qiagen, Hilden, Germany). miRNA was reverse transcribed using the miRCury LNA RT kit (Qiagen, Hilden, Germany) and amplification was performed using miRCury LNA SYBR green PCR kit in 96-well plates of an miRCury LNA miRNA custom PCR panel (Qiagen, Hilden, Germany). The following miRNA-specific primers were prespotted on the plate: hsa-miR-181b-5p, hsa-miR-126-5p, hsa-miR-30b-5p, hsa-miR-181a-5p, hsa-miR-26a-5p, hsa-miR-410-3p, hsa-miR-155-5p, hsa-miR-92a-3p, hsa-miR-19b-3p, hsa-let-7c-5p, hsa-let-7i-5p, hsa-miR-151a-3p, hsa-miR-98-5p, hsa-miR-103a-3p, hsa-miR-221-3p, hsa-miR-23a-3p, hsa-miR-574-3p, hsa-miR-1246, hsa-miR-146-5p, hsa-let-7d-3p, hsa-miR-222-3p and hsa-miR-143-3p and, as spike-in controls, uniSp6 and uniSp3. Selection of miRNAs for qRT-PCR with cells from 6 additional Donors was made for the top 3-5 miRNAs with the lowest *p*-value after NGS. In addition, we included also some miRNAs that were found to be relatively constant between different O_2_ conditions when analyzed via NGS (for instance hsa-miR-151a-3p, hsa-miR-221-3p NS hsa-miR-22-3p). Amplification was performed using a QuantStudio3 machine with the following temperature program: 2 min 95 °C for initial heat activation and 40 cycles of 10 s 95 °C for denaturation and 60 s 56 °C for annealing and extension followed by a 60–95 °C melting curve analysis.

### 4.7. RNA Sequencing (RNAseq) of Cellular mRNAs

Total RNA was isolated from attached cells after removing supernatants using the RNeasy mini plus kit (Qiagen, Hilden, Germany). Sequencing libraries for total RNA were prepared at the Core Facility Genomics, Medical University of Vienna, using the QuantSeq 3′ FWD protocol version 2 with unique dual indices (Lexogen, Vienna BioCenter), following the low input branch of the protocol. A total of 20 PCR cycles were used for library prep, as determined via qPCR according to the library prep manual. Libraries were quality-checked on a Bioanalyzer 2100 (Agilent) using a High-Sensitivity DNA Kit for correct insert size and quantified using Qubit dsDNA HS Assay (Invitrogen). Pooled libraries were sequenced on a NextSeq500 instrument (Illumina) in 1x75bp single-end sequencing mode. An average of 6 million reads per sample were generated. Reads in fastq format were generated using the Illumina bcl2fastq command line tool (v2.19.1.403). Reads were trimmed and filtered using cutadapt version 2.8 to trim polyA tails, remove reads with N’s and trim bases with a quality of less than 30 from the 3′ ends of the reads. On average, 4.8 million reads were left after this procedure. Trimmed reads in fastq format were aligned to the human reference genome version GRCh38 with Gncode 29 annotations using STAR aligner [53] version 2.6.1a in 2-pass mode. Raw reads per gene were counted with STAR.

### 4.8. Data Analysis of qRT-PCR Results and Statistical Evaluation

qPCR data were analyzed by normalizing Ct values towards spike-in controls, and as quantitative measure for relative expression, we used dCt = Ct (different O_2_ conditions)-Ct (21% O_2_), which is equivalent to −log(2) fold change (FC).

### 4.9. Bioinformatical Processing of Data

#### 4.9.1. Extracellular miRNAs

NGS sample data were processed according to the miRNA next-generation-sequencing discovery assay (miND) [51]. Differential expression of miRNAs in the endothelial secretome was analyzed by the established toolkit “edgeR” from NGS data [54]. Identified miRNAs found to be significantly changed in their abundance in the cell culture supernatants in response to different oxygen conditions were uploaded to the web-based platform miRNet 2.0 (https://www.mirnet.ca) in order to construct miRNA–target mRNA networks and to perform pathway enrichment analysis using Reactome database.

#### 4.9.2. Cellular mRNAs

Raw data of cellular mRNA gene expression as obtained from RNAseq analysis were analyzed using the R-packge “LIMMA” followed by gene set variation analysis (GSVA) [55]. A coexpression network was generated using weighted gene coexpression network analysis (WGCNA) [9] (see below), and modules of correlated genes with similar coexpression patterns were identified. Pathway enrichment analysis was performed on genes belonging to individual modules as well as correlation analysis in order to identify modules which were, in a statistical sense, significantly outstanding with a positive or negative correlation coefficient compared to all other treatment groups.

WGCNA was performed using variance-stabilized transformed counts matrices and the WGCNA package of R using signed networks, Pearson correlation and a beta of 8. The biologic context of the resulting modules was assessed using the ClusterProfiler package [56] and the molecular signature database, collection Geneontology—biological process (GoBP) [57].

miRNA–RNA interactions were determined using the iTALK package of R and the molecular signature database (collection C2) to determine mRNA targets of miRNAs [11,57].

## 5. Conclusions

This pilot study let us identify some candidate miRNAs that have the potential to serve as indicators of the (lung) microvascular endothelium response to altered oxygen conditions and might mediate some of the downstream effects of these conditions on circulation and other organs. Owing to the rare availability of pure pulmonary microvascular endothelial cell preparations from healthy human lungs, the rather small sample size of this study does not allow for the unambiguous identification of miRNAs that function as biomarkers for different oxygen conditions. However, in the light of other data from previous studies, the predicted biological processes affected by these miRNAs fit into the picture. These results can and should be validated in future preclinical or clinical studies in order to better evaluate their potential as part of biomarker panels.

## Figures and Tables

**Table 1:** Fold change of secreted miRNAs with *p* < 0.05. as identified and quantified with NGS. Some of the data were verified with qRT-PCR.

O_2_ Condition	miRNA	NGS Results NGS (Donors 1–6)		miRNAs with *p* < 0.1 after qPCR Validation (Incl. Donors 7–12)
		Fold Change (Rel. to 21% O_2_)	*p*-Value (<0.05)	Fold Change (Rel. to 21% O_2_)
5% O_2_	hsa-miR-19b-3p	1.3386	0.0049	1.3068
	hsa-miR-222-3p	0.8383	0.0092	0.8405
	hsa-miR-23a-3p	1.2177	0.0145	1.080
	hsa-miR-26a-5p	0.8414	0.0185	0.8906
	hsa-let-7d-3p	0.7748	0.0197	1.119 *
	hsa-miR-21-3p	1.2995	0.0228	1.388
	hsa-miR-126-5p	0.8418	0.0294	1.0665
	hsa-miR-181c-3p	1.3927	0.0384	
	hsa-miR-1307-5p	1.3358	0.0392	
	hsa-miR-92a-3p	0.8453	0.0464	
10% O_2_	hsa-miR-4492	0.5117	0.0365	
95% O_2_	hsa-miR-181b-5p	0.6695	0.0037 (FDR = 0.096)	1.097 *
	hsa-miR-181a-5p	0.7929	0.0314	0.899
	hsa-miR-155-5p	0.7728	0.0322	1.033 *
	hsa-let-7c-5p	0.7914	0.0325	
0–21% O_2_	hsa-miR-30b-5p	1.9702	0.0036	2.1687
	hsa-miR-410-3p	0.5967	0.0067	0.993
	hsa-let-7i-5p	0.7756	0.0130	1.190 *
	hsa-miR-129-5p	2.7431	0.0171	Ct > 35
	hsa-miR-1304-3p	0.7181	0.0189	
	hsa-miR-125a-5p	1.3159	0.0229	0.956
	hsa-miR-654-3p	1.3576	0.0289	
	hsa-miR-224-5p	1.4676	0.0290	
	hsa-miR-92a-3p	0.8009	0.0339	
	hsa-miR-4497	0.6404	0.0344	
	hsa-miR-629-5p	0.6743	0.0377	
	hsa-miR-134-5p	1.2977	0.0388	
0–95% O_2_	hsa-let-7d-3p	0.7424	0.0026	0.8045
	hsa-miR-155-5p	0.7668	0.0110	0.7423
	hsa-miR-574-3p	1.2817	0.0154	1.1975
	hsa-miR-4492	0.5622	0.0298	
	hsa-miR-103a-3p	0.8102	0.0313	
	hsa-miR-23a-3p	1.2192	0.0338	1.018
	hsa-miR-301b-3p	0.4778	0.0339	
	hsa-miR-130a-3p	0.7906	0.0391	
	hsa-miR-7-5p	0.8296	0.0414	
	hsa-miR-26b-5p	0.8316	0.0479	
	hsa-miR-98-5p	(0.9409)	(0.1476)	(0.8409)

* NGS results not confirmed.

**Table 2:** Pathways associated with individual identified miRNAs and their targets as listed in the Reactome database.

O_2_ Condition	miRNA	Affected Pathways via Target Genes (Reactome Database)
5% O_2_	hsa-miR-19b-3p (↑)	Signaling by TGFßR, ErbB, SCF-KIT, Wnt. VEGFR-mediated cell proliferation
	hsa-miR-222-3p (↓)	Cell senescence, PI3K/Akt activation, cell cycle, signaling by SCF-KIT, DAP12, FGFR
	hsa-miR-23a-3p (↑)	IL6-signaling, ATF4 activated genes, signaling by SCF-KIT, EGFR, PI3K/Akt
	hsa-miR-26a-5p (↓)	Cellular response to stress, gene expression, cellular senescence, cell cycle, DNA damage/telomer stress induction
	hsa-let-7d-3p (↓)	Anti-cell proliferation, granulopoiesis, T-cell differentiation, cell division (not annotated to exosomes)
	hsa-miR-21-3p (↑)	Methylation, VEGFA-VEGFR2 signaling, Rho-GTPase signaling, transcriptional regulation by TP53, regulation of actin dynamics
	hsa-miR-126-5p (↓)	IL-6 signaling, collagen degradation, miRNA biogenesis, PIP3K/Akt, NOTCH
	hsa-miR-181c-3p (↑)	Calcium activated K ^+^ channels, fibrin clot formation, detoxification of ROS, smooth muscle contraction
	hsa-miR-1307-5p (↑)	Cell–extracellular matrix interaction, mRNA splicing, translocation of GLUT4 to plasma membrane, VEGFA-VEGFR2 signaling, apoptosis, synthesis of very long fatty acids-CoA
	hsa-miR-92a-3p (↓)	Cell cycle, gene expression, translation
10% O_2_	hsa-miR-4492 (↓)	Cellular senescence (oxidative stress induced), DAP12 interactions, heme degradation, nuclear events
95% O_2_	hsa-miR-181b-5p (↓)	Cellular senescence and response to stress, DNA fragmentation, apoptosis, signaling by ERBB, NOTCH, calcium-dependent events, IL-1 signaling, interferon signaling, TLR 7/8
	hsa-miR-181a-5p (↓)	Cell response to stress, signaling by EGFR, ERBB, FGFR, PDGF, RAF/MAPK cascade, signaling by interleukins, immune system, DNA fragmentation
	hsa-miR-155-5p (↓)	NFKB, B-cell, T-cell receptor, p53, NOD-like receptor, TLR, SMAD, and interleukin (-6) signaling; cellular response to stress, cellular senescence
	hsa-let-7c-5p (↓)	IL-6, SMAD, SCF-KIT, PI3K/Akt, p53, and NOTCH signaling, respiratory electron transport, cellular senescence, platelet activation, aggregation and degranulation, immune system, ATF6-dependent activation of chaperons
0–21% O_2_	hsa-miR-30b-5p (↑)	NOTCH, SMAD, TGFßR, and IFNA signaling, intrinsic pathway of apoptosis, cellular senescence, DNA repair, response to oxidative stress
	hsa-miR-410-3p (↓)	Beta-catenin phosphorylation cascade, platelet activation, signaling and aggregation, prolonged ERK activity, CaM pathway, calmodulin induced events
	hsa-let-7i-5p (↓)	Platelet degranulation, DNA damage/stress-induced senescence, cytokine signaling in immune system, ATF4-activated genes, IFN signaling, PERK regulated gene expression, intrinsic pathway of apoptosis, chromatin organization
	hsa-miR-129-5p (↑)	RSK activation, sphingolipid metabolism, circadian clock, ERK/MAPK targets, immune system, GABA/NMDA receptor activation, eNOS activation, oxidative stress-induced senescence
	hsa-miR-1304-3p (↓)	mRNA splicing, ER quality control, biosynthesis of N-glycan precursors, calnexin/calreticulin cycle, Asn-linked N-glycosylation, N-glycan trimming in ER, actin dynamics
	hsa-miR-125a-5p (↑)	Glycolysis, NGF, SCF-KIT, PDGF, Akt, CTLA4, NOTCH, VEGF, FGFR, TLR, and MAPK signaling
	hsa-miR-654-3p (↑)	Akt-, IFNG-, FGFR-, EGFR-, PDGF-signaling
	hsa-miR-224-5p (↑)	SCF-KIT, EGFR, NGF, VEGF, DAP12, Akt, and MAPK signaling: Rho GTPases activate PAKs, intrinsic pathway of apoptosis
	hsa-miR-92a-3p (↓)	Cell cycle, NOTCH-, Wnt-signaling
	hsa-miR-4497 (↓)	Rho-GTPases, VEGF and leptin-signaling; response to hypoxia, smooth muscle contraction, platelet degranulation, DNA repair, cytokine signaling in immune system
	hsa-miR-629-5p (↓)	HATs deacetylate histones, chromatin organization, DNA methylation, NLRP3 inflammasome, tight junctions, cell cycle, apoptosis
	hsa-miR-134-5p (↑)	Telomere maintenance and extension, cell response to hypoxia, VEGF and interleukin-(-6) signaling, NLRP3 inflammasome, p38MAPK, SOS-mediated signaling, oxygen-dependent proline hydroxylation
0–95% O_2_	hsa-let-7d-3p (↓)	Transcription, cGMP effects, metabolism of non-coding RNAs, platelet homeostasis
	hsa--miR-155-5p (↓)	See above (as with 95% O_2_)
	hsa-miR-574-3p (↑)	Regulation of HIFs, PCP/CE pathway, DSCAM and LICAM interactions, inactivation of Cdc42 and Rac, RhoGTPases activation of NOX, TRAF-dependent IRF activation, Rho GTPases activate IQGAPs
	hsa--miR-4492 (↓)	Crosslinking of collagen fibrils, heme degradation, nuclear events, GPVI-mediated activation cascade
	hsa-miR-103a-3p (↓)	siRNA biogenesis, Ca^2+^ pathway, G-protein activation, opoid signaling, insulin processing, cell cycle
	hsa-miR-23a-3p (↑)	IL-6 signaling, ERBB, SCF-KIT, EGFR, FGFR, PI3K/Akt, and SMAD signaling, death receptor signaling, Ca^2+^ pathway
	hsa-miR-301b-3p (↓)	NF-KB activation for survival, oxygen-dependent proline hydroxylation, Ca^2+^ pathways, regulated necrosis, IRAK2-mediated activation of TAK1, insuline receptor recycling
	hsa-miR-130a-3p (↓)	O_2_-dependent proline hydroxylation, Ca^2+^ pathways, Wnt, NOTCH, and BMP signaling, glycogen synthesis, circadian clock, regulation of necroptotic cell death, IRAK1 recruits IKK
	hsa-miR-7-5p (↓)	IRS-mediated signaling, SCF-KIT, EGFR, ERK, NGF, ERBB, Akt, FGFR, and VEGF signaling, immune system, IL-2 signaling, apoptosis
	hsa-miR-98-5p (↓)	IL-6 and IFN-signaling, cellular stress response and senescence, HATs acetylate histones, glucose transport

Arrow up = upregulated; arrow down = down regulated.

**Table 3:** Correlation coefficients of individual modules from quantified miRNAs after WGCNA.

Module	10% O_2_	21% O_2_	5% O_2_	95% O_2_	0–21% O_2_	0–95% O_2_
**M13**	−0.125	−0.007	0.059	−0.082	−0.204	0.359
	*p* = 0.663	*p* = 0.980	*p* = 0.837	*p* = 0.777	*p* = 0.473	*p* = 0.193
**M8**	−0.243	0.154	0.393	−0.062	−0.294	0.051
	*p* = 0.391	*p* = 0.590	*p* = 0.149	*p* = 0.829	*p* = 0.294	*p* = 0.861
**M0**	−0.227	0.203	0.156	0.042	−0.271	0.098
	*p* = 0.424	*p* = 0.476	*p* = 0.586	*p* = 0.886	*p* = 0.334	*p* = 0.734
**M2**	−0.192	0.157	0.196	0.025	−0.252	0.066
	*p* = 0.502	*p* = 0.583	*p* = 0.492	*p* = 0.932	*p* = 0.372	*p* = 0.819
**M4**	0.260	−0.014	0.0278	−0.0623	−0.166	−0.045
	*p* = 0.356	*p* = 0.961	*p* = 0.924	*p* = 0.828	*p* = 0.562	*p* = 0.875
**M7**	0.138	0,028	0.061	−0.024	−0.208	0.004
	*p* = 0.629	*p* = 0.924	*p* = 0.833	*p* = 0.935	*p* = 0.465	*p* = 0.988
**M14**	−0.173	−0.029	−0.008	0.325	−0.136	0.021
	*p* = 0.546	*p* = 0.920	*p* = 0.976	*p* = 0.243	*p* = 0.636	*p* = 0.0.942
**M6**	−0.243	0.069	0.113	−0.049	0.075	0.037
	*p* = 0.390	*p* = 0.811	*p* = 0.695	*p* = 0.864	*p* = 0.796	*p* = 0.899
**M5**	−0.020	−0.007	−0.006	0.090	0.051	−0.109
	*p* = 0.945	*p* = 0.982	*p* = 0.985	*p* = 0.754	*p* = 0.859	*p* = 0.704
**M9**	0.000	0.000	−0.054	0.064	0.030	−0.041
	*p* = 0.999	*p* = 0.999	*p* = 0.853	*p* = 0.825	*p* = 0.917	*p* = 0.888
**M1**	−0.022	−0.015	0.044	−0.013	0.023	−0.017
	*p* = 0.940	*p* = 0.957	*p* = 0.879	*p* = 0.964	*p* = 0.936	*p* = 0.953
**M10**	−0.006	−0.025	0.046	−0.034	0.011	0.007
	*p* = 0.984	*p* = 0.931	*p* = 0.873	*p* = 0.907	*p* = 0.970	*p* = 0.979
**M12**	0.018	−0.012	0.005	0.025	−0.034	−0.002
	*p* = 0.951	*p* = 0.966	*p* = 0.986	*p* = 0.931	*p* = 0.907	*p* = 0.995
**M11**	0.009	0.000	0.023	−0.027	−0.023	0.017
	*p* = 0.975	*p* = 0.999	*p* = 0.936	*p* = 0.925	*p* = 0.937	*p* = 0.952
**M3**	0.027	0.036	0.004	−0.018	−0.034	−0.015
	*p* = 0.926	*p* = 0.902	*p* = 0.988	*p* = 0.949	*p* = 0.907	*p* = 0.960

**Table 4:** Module assignment of NGS-identified miRNAs and network connectivity.

O_2_ Condition	miRNA	Connectivity			
		Module	k_Total_	k_within_	K_within norm_
5% O_2_	hsa-miR-19b-3p	M1	26.839	15.669	0.948
	hsa-miR-222-3p	M3	22.589	7.305	0.504
	hsa-miR-23a-3p	M1	9.843	6.341	0.384
	hsa-miR-26a-5p	M9	7.440	4.546	0.618
	hsa-let-7d-3p	M3	10.311	4.149	0.286
	hsa-miR-21-3p	M3	17.604	9.434	0.651
	hsa-miR-126-5p	M3	2.830	1.143	0.079
	hsa-miR-181c-3p	M11	0.259	0.002	0.000
	hsa-miR-1307-5p	M1	7.606	4.265	0.258
	hsa-miR-92a-3p	M3	31.065	14.481	1.000
10% O_2_	hsa-miR-4492	M3	23.365	7.392	0.510
95% O_2_	hsa-miR-181b-5p	M1	12.731	7.049	0.426
	hsa-miR-181a-5p	M10	0.852	0.412	0.088
	hsa-miR-155-5p	M9	4.433	2.099	0.285
	hsa-let-7c-5p	M3	15.407	6.221	0.430
0–21% O_2_	hsa-miR-30b-5p	M5	1.718	1.051	0.361
	hsa-miR-410-3p	M14	2.224	1.517	0.578
	hsa-let-7i-5p	M11	0.673	0.068	0.026
	hsa-miR-129-5p	M12	0.758	0.429	0.246
	hsa-miR-1304-3p	M6	0.831	0.122	0.009
	hsa-miR-125a-5p	M3	0.188	0.057	0.004
	hsa-miR-654-3p	M10	3.774	2.657	0.568
	hsa-miR-224-5p	M9	0.452	0.188	0.026
	hsa-miR-92a-3p	M3	31.065	14.481	1.000
	hsa-miR-4497	M3	2.830	1.143	0.079
	hsa-miR-629-5p	M3	10.974	6.603	0.456
	hsa-miR-134-5p	M10	4.226	3.025	0.647
0–95% O_2_	hsa-let-7d-3p	M3	10.311	4.149	0.286
	hsa-miR-155-5p	M9	4.433	2.099	0.285
	hsa-miR-574-3p	M0	1.747	0.174	0.014
	hsa-miR-4492	M3	23.365	7.392	0.510
	hsa-miR-103a-3p	M5	3.904	2.828	0.972
	hsa-miR-23a-3p	M1	9.843	6.341	0.384
	hsa-miR-301b-3p	M5	0.940	0.322	0.111
	hsa-miR-130a-3p	M1	3.773	2.369	0.143
	hsa-miR-7-5p	M1	26.829	13.911	0.842
	hsa-miR-26b-5p	M5	3.067	2.454	0.843
	hsa-miR-98-5p	(M0)	0.378	0.082	0.007

**Table 5:** Correlation coefficients of individual modules from quantified cellular mRNA after WGCNA. Significant correlation coefficients (*p* < 0.05) are highlighted in red (=positive correlation; *p* < 0.0001: darker red) and blue (=negative correlation; *p* < 0.0001 darker blue).

Module	10% O_2_	21% O_2_	5% O_2_	95% O_2_	0–21% O_2_	0–95% O_2_	Module	10% O_2_	21% O_2_	5% O_2_	95% O_2_	0–21% O_2_	0–95% O_2_
**M87**	0.17	−0.16	0.39	−0.48	0.18	−0.05	**M39**	−0.04	−0.21	0.00	0.51	0.13	−0.16
**M16**	0.09	0.05	−0.03	−0.35	0.18	0.19	**M40**	0.11	−0.30	−0.32	0.41	0.20	−0.17
**M73**	0.19	0.04	−0.05	−0.26	0.09	−0.09	**M67**	0.20	−0.32	−0.02	0.39	−0.07	−0.23
**M90**	0.14	0.07	0.13	−0.33	0.10	0.02	**M72**	0.00	0.11	−0.22	0.45	−0.25	0.30
**M12**	0.03	0.18	0.04	−0.29	−0.03	0.04	**M80**	0.11	−0.16	−0.11	0.03	0.22	0.30
**M70**	0.18	0.09	0.03	−0.25	−0.03	−0.01	**M48**	0.13	−0.02	−0.11	0.14	−0.19	0.34
**M82**	0.09	0.01	0.28	−0.27	0.09	0.20	**M89**	0.21	−0.07	−0.08	0.05	−0.03	0.17
**M71**	0.20	0.02	0.18	−0.15	−0.02	−0.01	**M44**	−0.09	0.32	0.21	0.08	−0.10	−0.10
**M30**	0.14	0.00	0.26	−0.13	−0.05	0.13	**M76**	−0.19	0.31	0.09	0.03	0.18	0.07
**M6**	0.04	0.04	0.19	−0.11	−0.07	0.16	**M52**	−0.04	0.03	0.13	0.29	−0.03	−0.02
**M8**	0.19	0.05	0.24	−0.30	0.37	−0.21	**M86**	−0.01	−0.05	0.21	0.14	−0.11	−0.01
**M84**	0.16	0.15	0.10	−0.23	0.28	−0.23	**M28**	0.13	0.13	0.28	−0.06	−0.32	0.11
**M47**	0.05	−0.04	0.08	−0.34	0.45	0.23	**M9**	0.21	0.10	0.03	0.08	−0.23	0.03
**M63**	−0.02	−0.18	0.18	−0.19	0.33	0.11	**M27**	0.38	−0.23	0.34	−0.11	−0.13	−0.15
**M91**	−0.01	−0.16	0.46	−0.18	0.56	−0.39	**M35**	0.20	0.03	0.34	−0.27	−0.33	0.02
**M54**	−0.19	0.10	0.16	0.04	0.39	−0.33	**M93**	0.18	0.02	0.28	−0.23	−0.16	−0.04
**M41**	−0.18	0.12	−0.05	0.03	0.32	−0.06	**M62**	0.25	−0.48	0.12	−0.15	0.02	0.16
**M79**	−0.10	0.03	−0.06	0.15	0.38	−0.17	**M69**	0.10	−0.16	−0.17	−0.15	0.17	0.11
**M19**	0.17	−0.25	0.04	−0.08	0.44	−0.06	**M81**	0.00	−0.17	0.03	−0.32	0.15	0.06
**M64**	0.03	−0.25	−0.08	−0.02	0.54	−0.05	**M42**	0.08	0.21	−0.01	0.10	−0.46	−0.20
**M21**	0.22	−0.10	−0.09	−0.08	0.30	−0.02	**M14**	0.07	0.31	−0.21	0.15	−0.25	−0.26
**M55**	0.13	−0.01	−0.22	−0.04	0.44	−0.14	**M32**	0.12	0.37	−0.24	−0.01	−0.23	−0.20
**M85**	−0.25	0.14	−0.28	0.43	−0.03	0.02	**M66**	0.10	0.27	0.18	−0.21	−0.23	−0.35
**M15**	−0.11	−0.14	0.03	0.34	−0.19	0.06	**M17**	0.06	0.26	0.22	−0.18	−0.41	−0.04
**M31**	−0.33	−0.14	−0.01	0.29	0.02	0.06	**M57**	0.11	0.22	0.29	−0.02	−0.51	−0.14
**M60**	−0.29	0.07	0.02	0.26	−0.02	0.00	**M58**	−0.06	0.16	−0.07	−0.17	0.09	−0.09
**M43**	−0.30	0.13	−0.21	0.39	−0.52	0.01	**M18**	−0.06	0.19	−0.22	0.03	0.06	−0.14
**M29**	0.04	−0.02	0.05	0.24	−0.50	0.16	**M53**	0.00	0.10	−0.13	0.01	0.18	−0.14
**M25**	0.10	−0.01	−0.06	0.31	−0.42	−0.05	**M34**	0.10	0.04	0.10	0.14	−0.15	−0.26
**M50**	−0.01	0.11	−0.01	0.15	−0.46	0.01	**M46**	0.23	−0.10	0.10	0.17	−0.13	−0.15
**M23**	−0.03	0.05	−0.13	0.10	−0.51	0.28	**M88**	0.00	−0.15	0.04	0.09	0.15	−0.04
**M49**	−0.12	−0.01	−0.11	0.20	−0.40	0.04	**M3**	0.03	0.02	0.04	0.00	−0.07	0.01
**M92**	−0.14	0.05	−0.07	0.16	−0.52	0.16	**M0**	0.05	−0.07	0.06	0.08	−0.04	−0.02
**M51**	−0.33	0.08	−0.40	0.19	0.00	0.18	**M2**	0.01	−0.07	0.03	0.06	−0.01	0.02
**M68**	−0.15	0.09	−0.38	−0.06	0.11	0.12	**M20**	0.00	−0.05	0.02	0.01	−0.03	0.04
**M65**	−0.42	−0.12	0.03	−0.05	0.06	0.30	**M78**	0.10	−0.15	0.11	−0.09	0.07	−0.02
**M4**	−0.31	0.04	−0.15	0.05	−0.17	0.22	**M5**	0.00	−0.02	0.02	−0.01	0.07	−0.03
**M74**	−0.23	−0.12	−0.03	−0.01	−0.01	0.13	**M36**	0.10	0.00	0.05	−0.11	0.03	−0.01
**M94**	−0.25	−0.04	−0.10	0.13	−0.11	0.07	**M1**	0.05	0.04	0.05	−0.12	0.10	−0.09
**M37**	−0.11	−0.43	−0.22	0.28	0.24	0.11	**M10**	0.07	−0.01	0.10	−0.05	0.06	−0.11
**M77**	−0.16	−0.39	−0.14	0.17	0.29	0.28	**M61**	0.16	0.38	−0.25	−0.19	−0.14	0.16
**M13**	−0.16	−0.12	−0.15	0.00	0.28	−0.02	**M22**	−0.18	0.04	0.12	0.10	−0.29	0.22
**M59**	−0.22	−0.20	−0.17	−0.14	0.48	0.18	**M75**	−0.22	0.23	0.13	−0.09	−0.17	0.28
**M33**	0.06	−0.33	−0.12	0.34	−0.30	0.10	**M56**	−0.06	0.24	−0.06	−0.21	−0.33	0.25
**M38**	0.06	−0.33	−0.01	0.37	−0.25	−0.02	**M7**	−0.04	0.04	0.03	−0.13	−0.28	0.22
**M26**	0.13	−0.33	−0.28	0.54	−0.10	0.08	**M11**	0.10	0.20	0.05	−0.16	−0.23	−0.03
**M83**	−0.10	−0.22	−0.19	0.53	−0.12	−0.01	**M24**	−0.01	0.08	−0.07	−0.09	−0.10	0.11
**M45**	−0.16	0.22	−0.05	−0.06	−0.15	0.04							

**Table 6:** Enriched functional terms of modules with highly correlated gene expression.

5% O_2_		
positive correl.	M87	Microtubule cytoskeleton organization, cell–cell junction assembly, macroautophagy, store-operated calcium entry
	M91	Regulation of ROS biosynthesis, cytokine production, mitochondrial calcium homeostasis
negative correl.	M51	Membrane organization, cytokinesis, cell division
		
10% O_2_		
negative correl.	M65	Protein polyubiquitination, cell division
		
0–21% O_2_		
positive correl.	M91	(see above)
	M54	Intertypic cell–cell adhesion, positive regulation of stress fiber assembly, reactive nitrogen species metabolism, IL-1, IFNγ production
	M19	Glycosylation, lipid biosynthesis, positive regulation of IKß kinase, NFKB signaling, cell-matrix adhesion
	M64	VEGF/VEGFR1 pathway, angiopoietin receptor pathway, PI3K/Akt signaling, NTRK2/TRKB signaling
	M59	Glycosphingolipid biosynthesis, TOLL-like receptor signaling, apoptosis
negative correl.	M43	Protein sumoylation, ribosome biogenesis
	M29	Epigenetic regulation of gene expression, base excision repair, telomere maintenance, lipid homeostasis
	M25	Mitochondrial outer-membrane permeabilization, fatty acid beta oxidation
	M50	Base excision repair, thrombin PAR1 pathway
	M23	Vesicle organization, cellular senescence
	M49	P53 signaling pathway, protein targeting to lysosome, respiration electron tramsport
	M92	Telomere maintenance via telomere lengthening, chaperone mediated protein folding, gene silencing by RNA
	M42	Nucleotide excision repair, receptor mediated endocytosis
	M17	Proteasome mediated ubiquitin-dependent protein catabolic process, protein exit from ER
	M57	Negative regulation of TOR and TORC1 signaling, signaling by FGFR, peroxisome
		
95% O_2_		
positive correl.	M85	Response to ionizing radiation, transcriptional activation of mitochondrial biogenesis, respiratory electron transport
	M43	(see above)
	M26	DNA damage/telomere stress-induced senescence, negative regulation of cell development, regulation of oxidative stress-induced intrinsic apoptotic signaling
	M83	Response to oxidative stress, DNA damage response, DNA integrity checkpoint signaling, negative regulation of mitochondrial cell cycle, transcriptional regulation by TP53
	M39	Negative regulation of developmental growth, positive regulation of fat cell differentiation, negative regulation of fibroblast prolifertation
	M40	Protein mono-, polyubiquitiniation, protein catabolic process, negative regulation of NOTCH signaling
	M67	Lipid homeostasis, DNA repair, DNA replication fidelity, telomere maintenance, cholesterol biosynthesis
	M72	Regulation of extrinsic apoptosis, DNA repair
negative correl.	M87	(see above)
		
0–95% O_2_		
negative correl.	M91	(see above)

**Table 7:** Pathways related to target genes conversely altered in HMVEC-Ls and secreted miRNAs (iTALK).

	95% O_2_	0–21% O_2_
miRNAs and Target genes altered in HMVEC-L	hsa-miR-155-5p_(TP53INP1, RAB11FIP2, TAB2, PPM1D, RGP1,TRPS1, ANTXR2, CEBPB, SIPR1) hsa-miR-181a-5p (ZBTB43. CCNK, PRTG, SLC7A11, RLIM, TAB2, BCL2, IPO7, REPS2, N4BP2, DNAJA4, PTBP3, COX15, PARM1, AKIRIN1, CREB1, OGFRL1, NCOA2, TNRC6B, ZDHHC7, MTURN, S1PR1, CREBRF, MTPN, SSB, PRTG) hsa-let-7c-5p (PRTG, MASP1, CEMIP2, SFSWAP, DDI2, SEMA4C, CPSF4, LTN1, TAF98, NIPA1, INTS2, NKAPD1, KIAA0930, MEF2D, BCL2L1, SLF2, NCOA1)	hsa-miR-30b-5p hsa-miR-129-5p hsa-miR-224-5p hsa-miR-125a-5p hsa-miR-654-3p hsa-miR-134-5p hsa-miR-92a-3p hsa-let-7i-5p hsa-miR-629-5p hsa-miR-1304-3p hsa-miR-410-3p corresponding target genes altered in mother cell (see Supplemental Data S4)
Signaling pathways	Intrinsic apoptosis Cell response to (chemical stress) ESR-mediated signaling KEAP1-NFE2L2 pathway IL4 and IL13 signaling NLRP1 inflammasome Transcriptional activation of mitochondrial biogenesis Circadian clock	Regulation of MECP2 expression and activity RUNX1 regulates expression of tight junctions IL7 signaling Transcription of BIM

**Table 8:** Putative signaling pathways affected by cooperative action of miRNAs (Reactome database).

5% O_2_	0–21% O_2_
Signaling by ERBB2, FGFR, GAB1, DAP12, NOTCH, PDGF, NGF PIE/Akt activation Ca^2+^ pathway Cellular response to stress	Cellular response to stress Oxidative stress-induced senescence (mitotic) cell cycle Signaling by RhoGTPase, NOTCH, Wnt, EGFR, FGFR, DAP12 Chromatin modification Epigenetic regulation of gene expression VEGFR2-mediated cell proliferation Histone methylation, DNA damage/telomere stress-induced senescence Ca^2+^ pathway
95% O_2_	0–95% O_2_
Cellular response to stress (oxidative stress-induced) cellular senescence Negative regulation of rRNA expression Immune system Cytokine signaling (IL-6) Apoptosis Activated TLR4 signaling	Oxidative stress-induced senescence PIP3/Akt activation Immune system Ca^2+^ pathway TP53 regulation of metabolic genes DNA methylation VEGFA/VEGFR2 pathway

## Data Availability

Raw data are available upon request.

## Supplementary Materials

The following supporting information can be downloaded at: https://www.mdpi.com/article/10.3390/ijms25168798/s1.
