# Supplementary material for: Candidate Signature miRNAs from Secreted miRNAome of Human Lung Microvascular Endothelial Cells in Response to Different Oxygen Conditions: A Pilot Study
Source: Int J Mol Sci. 2024 Aug 13;25(16):8798. doi: 10.3390/ijms25168798 (PMC11354369; doi:10.3390/ijms25168798)
Supplement: Supplementary file 1 [file ijms-25-08798-s001.zip › Supplemental Data/Supplemental Data 5.pdf]

Donor characteristics of primary cells used in this study:

Donor	age	gender	ethnicity	analysis
1	44y	male	hispanic	NGS
2	50y	male	hispanic	NGS
3	75y	female	caucasian	NGS
4	65y	male	caucasian	NGS
5	50y	male	caucasian	NGS
6	33y	female	caucasian	NGS
7	43y	female	caucasian	qPCR
8	40y	male	hispanic	qPCR
9	65y	male	caucasian	qPCR
10	75y	male	hispanic	qPCR
11	66y	female	caucasian	qPCR
12	50y	male	hispanic	qPCR
