# Supplementary figures and images for: Candidate Signature miRNAs from Secreted miRNAome of Human Lung Microvascular Endothelial Cells in Response to Different Oxygen Conditions: A Pilot Study
Source: Int J Mol Sci. 2024 Aug 13;25(16):8798. doi: 10.3390/ijms25168798 (PMC11354369; doi:10.3390/ijms25168798)

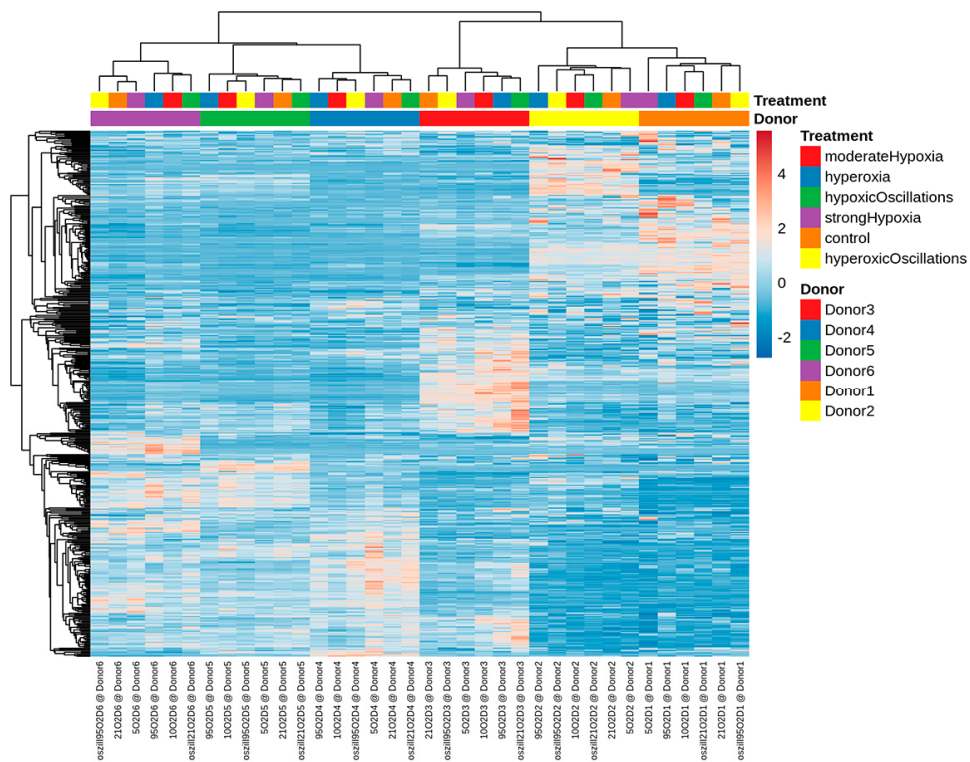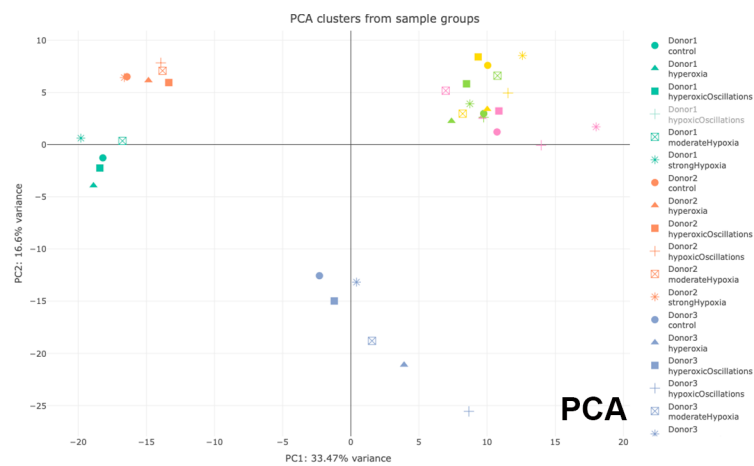

Supplemental Data:

Supplement: Supplementary file 1 [file ijms-25-08798-s001.zip › Supplemental Data/Supplemental Data 1B.pdf]
